# The Relationship between Accelerometry, Global Navigation Satellite System, and Known Distance: A Correlational Design Study †

**DOI:** 10.3390/s22093360

**Published:** 2022-04-27

**Authors:** Abdulmalek K. Bursais, Caleb D. Bazyler, Andrew R. Dotterweich, Adam L. Sayers, Mohammed S. Alibrahim, Anwar A. Alnuaim, Majed M. Alhumaid, Abdulrahman I. Alaqil, Ghareeb O. Alshuwaier, Jeremy A. Gentles

**Affiliations:** 1 Department of Physical Education, College of Education, King Faisal University, Al-Ahsa 31982, Saudi Arabia; malibrahim@kfu.edu.sa (M.S.A.); aalnuaim@kfu.edu.sa (A.A.A.); malhumaid@kfu.edu.sa (M.M.A.); aialaqil@kfu.edu.sa (A.I.A.); 2 Center of Excellence for Sport Science and Coach Education, East Tennessee State University, Johnson City, TN 37614, USA; bazylerc@mail.etsu.edu (C.D.B.); dotterwa@mail.etsu.edu (A.R.D.); sayersal@mail.etsu.edu (A.L.S.); gentlesj@mail.etsu.edu (J.A.G.); 3 Department of Sports Science and Clinical Biomechanics, University of Southern Denmark, 5230 Odense, Denmark; 4 Department of Exercise Physiology, College of Sport Sciences and Physical Activity, King Saud University, Riyadh 11451, Saudi Arabia; galshuwaier@ksu.edu.sa

**Keywords:** wearable technologies, accelerometers, GNSS, GPS, monitoring, training load, physical activity

## Abstract

**Background**: Previous research has explored associations between accelerometry and Global Navigation Satellite System (GNSS) derived loads. However, to our knowledge, no study has investigated the relationship between these measures and a known distance. Thus, the current study aimed to assess and compare the ability of four accelerometry based metrics and GNSS to predict known distance completed using different movement constraints. **Method:** A correlational design study was used to evaluate the association between the dependent and independent variables. A total of 30 physically active college students participated. Participants were asked to walk two different known distances (DIST) around a 2 m diameter circle (small circle) and a different distance around an 8 m diameter circle (large circle). Each distance completed around the small circle by one participant was completed around the large circle by a different participant. The same 30 distances were completed around each circle and ranged from 12.57 to 376.99 m. **Instrumentation:** Acceleration data was collected via a tri-axial accelerometer sampling at 100 Hz. Accelerometry derived measures included the sum of the absolute values of acceleration (SUM), the square root of the sum of squared accelerations (MAG), Player Load (PL), and Impulse Load (IL). Distance (GNSSD) was measured from positional data collected using a triple GNSS unit sampling at 10 Hz. **Results:** Separate simple linear regression models were created to assess the ability of each independent variable to predict DIST. The results indicate that all regression models performed well (R = 0.960–0.999, R^2^ = 0.922–0.999; RMSE = 0.047–0.242, *p* < 0.001), while GNSSD (small circle, R = 0.999, R^2^ = 0.997, RMSE = 0.047 *p* < 0.001; large circle, R = 0.999, R^2^ = 0.999, RMSE = 0.027, *p* < 0.001) and the accelerometry derived metric MAG (small circle, R = 0.992, R^2^ = 0.983, RMSE = 0.112, *p* < 0.001; large circle, R = 0.997, R^2^ = 0.995, RMSE = 0.064, *p* < 0.001) performed best among all models. **Conclusions:** This research illustrates that both GNSS and accelerometry may be used to indicate total distance completed while walking.

## 1. Introduction

Wearable technologies have become popular tools used in team and individual sports. Tracking player activity using these microtechnologies is an essential component of load monitoring [1]. Accelerometers and Global Navigation Satellite System (GNSS) devices have become some of the dominant wearable technologies used to monitor training-load in sport [2]. These technologies can be integrated or used separately to provide an indicator of the external work performed by athletes; consequently, practitioners may be better able to manage fatigue and direct adaptation. GNSS primarily measures horizontal displacement, while accelerometers primarily measure acceleration in single or multiple axes. The role of accelerometers and GNSS in load monitoring has received increased attention across a number of sports in recent years [3,4]. Despite this, the relationship between both technologies to quantify the same load is not well established.

Accelerometers are responsive motion sensors that measure the magnitude of acceleration in one or more axes. Accelerometers are valid and reliable instruments to measure training load in the field and in laboratory environments [5,6,7]. There is a growing body of literature that recognizes the ability of accelerometers to quantify the external demand of team and individual sports. For instance, the within and between device reliability of accelerometers has been established across a variety of movement demands in both laboratory and on-field conditions in Australian football [8]. Gentles et al. [9] found strong to nearly perfect correlations between accelerometry derived training load and session rating of perceived exertion (sRPE) (r = 0.84; *p* < 0.001) and total distance measured using GPS (r = 0.95; *p* < 0.001) among NCAA women’s soccer players [9]. Accelerometers have also been used to illustrate the differences in the activity profile between single and double match play in tennis [10]. In rugby, accelerometers outperformed GPS in quantifying positional (backs vs. forward) and halves (1st vs. 2nd) differences in player maximum mean movement [11]. Moreover, accelerometry has also been shown to be a valid assessment of a test designed to simulate basketball play, suggesting that accelerometers can be used to quantify the external demand of basketball [12].

Many accelerometry derived metrics have been used in the literature to quantify training load, including Body Load [13], Player Load [8], Force Load [14], Dynamic Stress Load [15], and Impulse Load [9]. Although Player Load is the most commonly reported measure in the literature [16], its potential to monitor training load has been questioned [17]. Player Load is the sum of the square root of the sum of absolute differences of acceleration divided by the device sampling frequency [17]. Therefore, Player Load does not represent the sum of all accelerations, and of the available accelerometry derived measures, it may not best represent training load [17]. Additionally, training-load could be misrepresented due to the inclusion of non-locomotor activities in Player Load [14]. Interestingly, different equations and descriptions for Player Load have also been reported in the literature [8,11,18]. Player Load has also been described as Body Load [13] and Acceleration Load [19]. To our knowledge, no study has compared different accelerometry derived measures when assessing training load, indicating a need for further investigation of accelerometry based measures of training load.

GNSS is an umbrella term that includes several different satellite networks including Global Positioning System (GPS), Global Navigation Satellite System (GLONASS), Galileo, and BeiDou. In sport, GNSS networks are used to provide information about a player’s position, velocity, and movement patterns on the field. Total distance and distance in speed zones are common variables used to monitor training loads. GNSS networks such as GPS have been shown to be valid indicators of distances of 40 m completed during different movement patterns but may not be a valid measure of shorter distances (less than 20 m) completed during high speed running, sprinting, and change of direction [20]. Higher sampling frequencies (5–10 Hz) have been shown to improve the accuracy of GPS [20,21], although some evidence suggests that increasing sampling frequency to 15 Hz does not improve accuracy when assessing distance completed during unstructured movements [22]. Assessing player position using GNSS networks may be influenced dramatically by the number and separation of satellites that are connected to the receiver. GNSS enables receivers to acquire signals from multiple satellite networks (e.g., GPS, GLONASS, Galileo, and BeiDou), increasing the number of available satellites. Combined satellite systems improve satellite geometry and resulting precision [23]. Dilution of Precision (DOP) is a description of satellite geometry. DOP is composed of two elements: horizontal dilution of precision (HDOP) and vertical dilution of precision (VDOP) (for more detail about DOP, see [23]). HDOP is one indicator of GNSS accuracy and is influenced by the separation of the satellites. HDOP values range from 0 to 50, with a value of less than 1 considered the ideal distribution of satellites. HDOP is low and precision is excellent when substantial distance exists between satellites, while HDOP is high and precision is poor when satellites are in close proximity. Additionally, indoor fields, stadiums with high walls or roofs, and cloudy weather are factors that can reduce the quality of GNSS data [4].

Recently, receivers capable of acquiring signals from multiple GNSS networks simultaneously (e.g., GPS, GLONASS, Galileo, and BeiDou) have enhanced the availability and signal strength of surrounding satellites [24]. Beato et al. [25] suggested that using multiple GNSS networks could explain the smaller bias (2.3 ± 1.1%) when measuring total distance during a sport-specific movement protocol [25] compared to the author’s previous research that used only GPS to detect total distance in a shuttle run over 5–20 m (2.53 ± 6.03%) [26]. Future research should compare the quality of data from single and multiple satellite systems in sport.

To date, several investigations have assessed the relationship between accelerometry and GNSS derived measures. A study by Polglaze et al. [27] found large to very large correlations between Player Load and total distance accumulated during men’s hockey practice (r = 0.742; *p* < 0.00001) and competition (r = 0.868; *p* < 0.00001) [27]. Additionally, a strong correlation was found between Player Load and total distance completed in men’s soccer training (r = 0.70; *p* < 0.01) [28], and a nearly perfect correlation (r = 0.95; *p* < 0.001) was found between Impulse Load and total distance in women’s soccer matches [9]. However, to our knowledge, no study has investigated the relationship between different accelerometry based metrics and GNSS with a known distance. Therefore, this study aimed to assess and compare the ability of four different accelerometry derived metrics and a triple GNSS to predict known distance completed under different movement constraints.

## 2. Materials and Methods

### 2.1. Experimental Approach for the Problem

**Figure 1:**
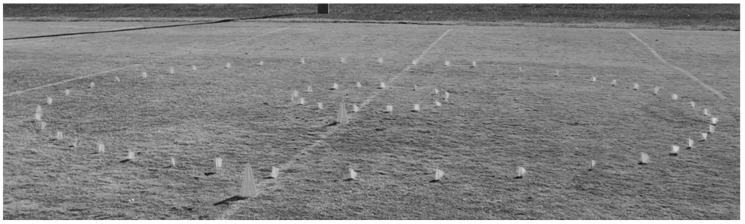
Design of the small and large circles.

A correlational design was used to assess the relationship between known distance (DIST) and total distance measured via GNSS and four accelerometry derived metrics. DIST was completed under two different movement constraints. Two courses, a small circle and a large circle, were designed on a grass field. Table 1 details the dimensions of each circle, and Figure 1 illustrates the course design. A measuring tape was used to measure the diameter of each circle, which was subsequently used to calculate circumference. Both circles were marked by flags to guide the walking path for subjects. Flags were approximately 5 cm in height to minimize interference with walking. Circles were used to limit the influence of initiating movement and braking associated with changing direction.

### 2.2. Participants

Thirty participants (height 176.8 ± 6.1 cm, weight 82.3 ± 12.8 kg) volunteered to participate in this study. All participants engaged in physical activity at least three times a week. This study was approved by the universities’ Institutional Review Board, and participants provided written consent for their involvement and video recording.

### 2.3. Procedures

Prior to beginning each course, participants were informed of the number of laps they were to complete around each course; participants also performed a familiarization trial prior to completing their trials. Following familiarization, each participant walked two different known distances (DIST), one distance around the small circle and a different distance around the large circle. Each distance completed around the small circle by one participant was completed around the large circle by a different participant. The same thirty distances were completed around each circle and ranged from 12.57 to 376.99 m. Table 2 details the number of laps and the total distance each participant completed. Participants were directed to walk at their normal speed and keep the flags between their feet during the walk to ensure each course was completed accurately. Laps were counted loudly by a research assistant during the trials. Each participant also wore a triaxial accelerometer and a triple GNSS sensor.

### 2.4. Instrumentation

Acceleration data were collected via a tri-axial accelerometer measuring the magnitude of acceleration in the three axes (*x* = anterior–posterior, *y* = medial–lateral, *z* = vertical) and sampling at 100 Hz (Zephyr^TM^ BioHarness v3, Zephyr Technology Corp., Annapolis, MD, USA). Using the chest strap provided by the manufacturer, the accelerometer was securely placed at the level of the xiphoid process, along the midsternal line. The sensor calibration and error model of the sensor is propriety to the manufacturer and has not been described by Zephyr^TM^. Four accelerometry derived metrics were used in this study; the formula for each accelerometry based metric is described in Table 3. To expedite data analysis, the beginning and end of each trial were marked by the participant tapping on the accelerometer four times.

A triple GNSS sensor sampling at 10 Hz and acquiring signals from GPS, GLONASS, and Galileo networks (Titan Sensors 2, Houston, TX, USA) was used to measure the distance covered by each participant (GNSSD). All trials were performed on an outside field, clear of large buildings, and with a clear sky to enhance satellite acquisitions. The number of satellites connected to the receiver during the trials ranged between 19–26. A previous study that used GNSS reported the horizontal dilution of precision (HDOP) 0.4 ± 0, while the satellites connected held between 18–20 [25]. The GNSS unit was activated 10–15 min prior to data collection and securely fixed to clothing on the back of participants at the base of the cervical spine between scapulae. Video was recorded (iPhone 6; 1080 p at 30 fps, Cupertino, CA, USA) and synced with GNSS data to verify the beginning and end of each trial.

### 2.5. Statistical Analyses

GNSS data and recorded video were uploaded and analyzed using Titan Sensors software (Titan Sync 3.0.0, 2019 and Titan Video 3.7.0, 2019). Accelerometry data were downloaded to OmniSense^TM^ Analysis (version 4.1.4; Zephyr Technology Corporation, Annapolis, MD, USA), then exported to Microsoft Excel 2019 (Microsoft Corporation, Redmond, WA, USA) for analysis. Data were log transformed using the natural logarithms (LN) of DIST, SUM, MAG, PL, IL, and GNSSD to reduce the nonuniformity of error [29]. Ten simple linear regression models were created to assess the ability of each independent variable (SUM, MAG, PL, IL, and GNSSD) to predict DIST completed during the small circle and large circle. Residual and Q-Q plots were used to ensure the assumptions of homoscedasticity and normality were not violated. All data were analyzed using the statistical software JASP (JASP, Version 0.12.2, Amsterdam, The Netherlands).

## 3. Results

**Figure 2:**
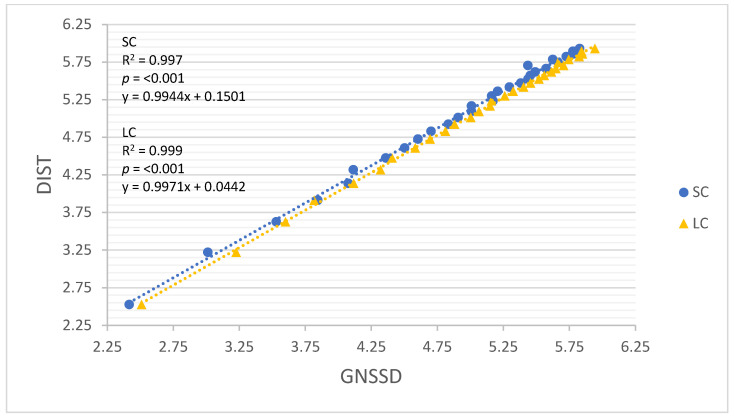
The relationship between log transformed DIST and GNSSD around the small circle and the large circle. SC = small circle, LC = large circle.

**Figure 3:**
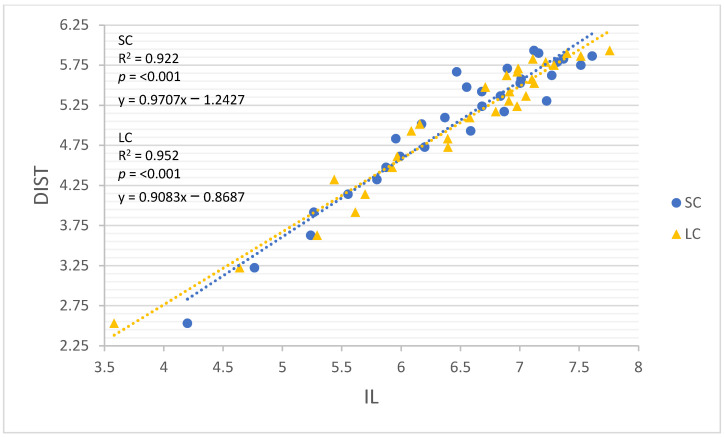
The relationship between log transformed DIST and IL around the small circle and the large circle. SC = small circle, LC = large circle.

**Figure 4:**
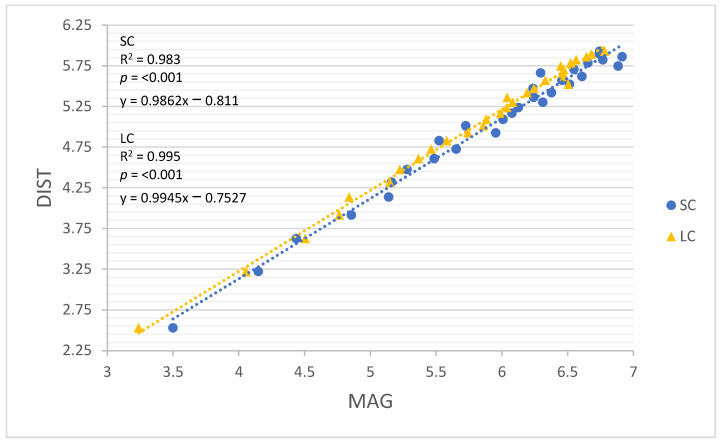
The relationship between log transformed DIST and MAG around the small circle and the large circle. SC = small circle, LC = large circle.

**Figure 5:**
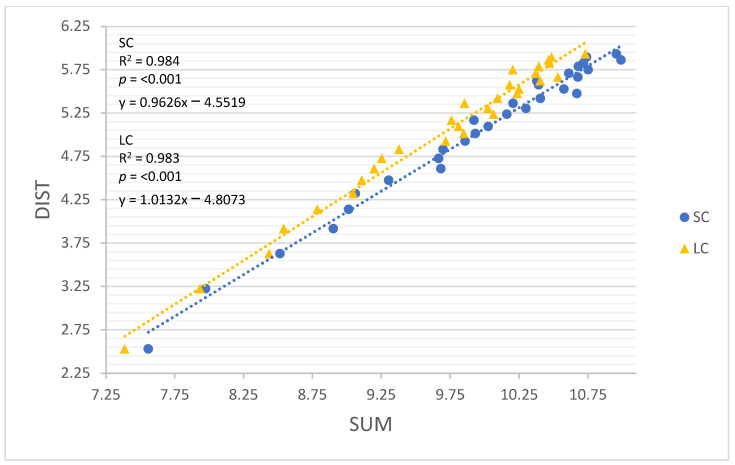
The relationship between log transformed DIST and SUM around the small circle and the large circle. SC = small circle, LC = large circle.

**Figure 6:**
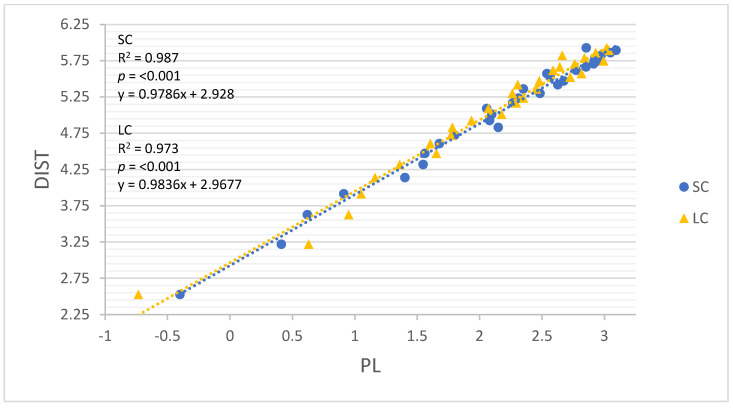
The relationship between log transformed DIST and PL around the small circle and the large circle. SC = small circle, LC = large circle.

All linear regression models performed well for both movement constraints (R = 0.960–0.999, R^2^ = 0.922–0.999; Root-mean-square error (RMSE) = 0.047–0.242, *p* < 0.001). The results of all linear regression models are detailed in Table 4, and each model is illustrated in Figure 2, Figure 3, Figure 4, Figure 5 and Figure 6. GNSSD (small circle, R = 0.999, R^2^ = 0.997, RMSE = 0.047, *p* < 0.001; large circle, R = 0.999, R^2^ = 0.999, RMSE = 0.027, *p* < 0.001) and the accelerometry derived metric MAG (small circle, R = 0.992, R^2^ = 0.983, RMSE = 0.112, *p* < 0.001; large circle, R = 0.997, R^2^ = 0.995, RMSE = 0.064, *p* < 0.001) performed best among all models. Table 5 (small circle) and Table 6 (large circle) provide details for each trial and includes laps and known distance completed, GNSS measured distance, and all accelerometry derived metrics.

## 4. Discussion

**Figure 7:**
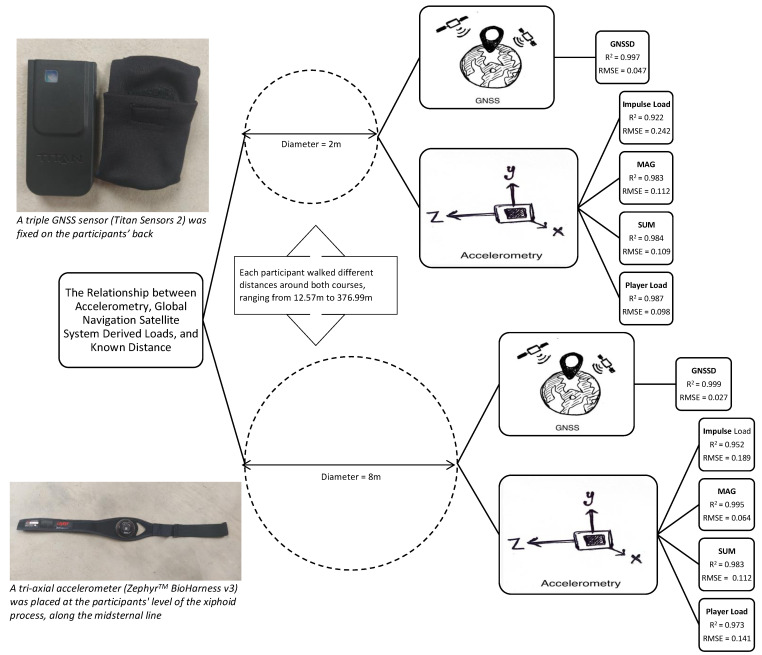
Demonstration and summary of the study results.

The purpose of this study was to assess and compare the ability of four different accelerometry derived metrics (IL, MAG, SUM, PL) and GNSS to predict a known distance completed using two movement constraints. A primary finding is that both GNSS and accelerometry derived measures are valid indicators of total distance when walking is performed around a small circle and a large circle. This may also suggest that both GNSS and accelerometry are similarly capable of quantifying the distance associated with sport-related training and competition under the current experimental conditions. The results of the study are demonstrated and summarized in Figure 7.

While all accelerometer derived measures performed well, MAG (small circle, R = 0.992, R^2^ = 0.983, RMSE = 0.112, *p* < 0.001; large circle, R = 0.997, R^2^ = 0.995, RMSE = 0.064, *p* < 0.001) and SUM (small circle, R = 0.992, R^2^ = 0.984, RMSE = 0.109, *p* < 0.001; large circle, R = 0.992, R^2^ = 0.983, RMSE = 0.112, *p* < 0.001) performed best among all accelerometry models. MAG and SUM include locomotor and non-locomotor activities. This outcome is contrary to Buchheit and Simpson [14], who proposed that using accelerometer-derived measures that exclude non-locomotor activities may be more useful [14]. However, this discrepancy could be attributed to the fact that little to no non-locomotor activity was included in this study, where sport includes a substantial quantity of both locomotive and non-locomotive activity. Our findings agree with previous suggestions that different accelerometry based metrics will not equally quantify training loads in sport-related events [17]. Further research is needed to determine which accelerometry derived metrics best quantify training load in sport. It is certainly possible that the simultaneous use of multiple accelerometry based load assessments is advantageous.

Unlike GNSS, accelerometers can be influenced by between-subjects’ variability in loading patterns (e.g., stride characteristics) [30]. However, in this study and others [9,27,28], strong relationships have been found between accelerometry derived loads and total distance, despite different participants completing various distances. Much of the criticism that PL has attracted relates to calculating workloads by summing the rate of change in accelerations instead of the absolute value of accelerations [17]. However, in this study, although PL did not perform best among the accelerometry derived measures, its potential to detect training load was encouraging (small circle, R = 0.994, R^2^ = 0.987, RMSE = 0.098, *p* < 0.001; large circle, R = 0.987, R^2^ = 0.973, RMSE = 0.141, *p* < 0.001). Nonetheless, it may be important to address how PL would perform if repeated changes of direction were included, given that PL only increases with changes in acceleration. In accordance with the present results, a previous study demonstrated that the accelerometry derived metric average force (the product of the participant’s body mass and MAG) was a better indicator of running demands compared to PL [12]. Future studies should investigate what, if any, advantages MAG or other accelerometry based measures may provide compared to PL.

Previous research has indicated that, independent of movement velocity (i.e., walk, jog, run, sprint), rapid directional change degrades GNSS accuracy. For instance, GNSS may underestimate distance during shuttle trials (−2.16 ± 3.84%) and overestimate distance during exercise completed on curvilinear tracks (2.99 ± 2.96%) [31]. However, the development of multiple GNSS technology may explain the high level of accuracy found in this study, which included two different curvilinear conditions (small circle, R = 0.999, R^2^ = 0.997, RMSE = 0.047, *p* < 0.001; large circle, R = 0.999, R^2^ = 0.999, RMSE = 0.027, *p* < 0.001). Despite these promising results, questions remain about multiple GNSS system accuracy when measuring the distance completed in sport-related movements, where many changes of direction are required, and movement velocity is often higher than that used in this study.

This investigation demonstrates that multiple GNSS systems and several accelerometry derived metrics can indicate total distance completed while walking. However, a host of questions remain regarding the potential advantages associated with these technologies to quantify training loads and detect events (e.g., contact, jumps, sprinting) in sport. In this study, total distance was the only load accounted for, and only locomotor movements were included. Of course, in sport, different factors can influence training loads (e.g., acceleration, deceleration, jumping), and locomotor and non-locomotor movements will be performed. More broadly, further investigation is needed to assess the ability of GNSS and accelerometry derived metrics to measure training loads that include different movements and events such as running, sprinting, change of direction, jumping, collision, and kicking.

Although this study demonstrated that GNSS and accelerometry derived measures are valid indicators of total distance, three important limitations to this study should be considered. First, while walking was performed around circles to limit the influence of initiating movement and braking associated with changing direction and total distance was the only variable assessed, caution should be used when applying the current results to other activities. Second, participants were asked to walk at their natural pace, and no method has been used to standardize the walking speed, in which differences in the velocity rate between participants might induce some variation. Third, while the investigators were precise during course set-up and the participants followed the instructions closely, the actual distances completed by the participants were likely different than planned; albeit these differences were probably very small. Future research should investigate whether training-load quantification is enhanced using a combination of GNSS and accelerometry, or whether a single sensor, GNSS, or accelerometer is adequate to quantify training loads in sports that often include changes of direction, jumping, contact, and straight-line movement.

## 5. Conclusions

This is the first study to investigate the ability of four different accelerometry derived metrics and a triple GNSS to predict known distance. Linear regression analysis revealed that GNSSD, IL, MAG, SUM, and PL could indicate total distance completed while walking. The findings will be of interest to researchers and sports scientists to investigate whether GNSS and accelerometry are equally capable of quantifying training loads associated with sport-related training and competition. More research using controlled trials are needed to compare these technologies to detect sports events (e.g., contact, jumps, sprinting) and quantify training loads associated with acceleration, deceleration, and directional change, which are considered crucial characteristics of match play in some sports. Another possible area of future research would be to investigate whether training-load quantification is enhanced using a combination of GNSS and accelerometry, or whether a single sensor, GNSS, or accelerometer is adequate to quantify training loads in sports, considering that not all teams can afford the high-cost of both technologies.

## 6. Practical Application

GNSS and accelerometers seem similarly capable of quantifying distance while walking. This may have implications for sport-related training and competition, but future research should investigate whether both sensor types perform comparably well during sport related activities that include mixed movement types (i.e., changes of direction, running, jumping, etc.). While sport coaches and athletes should use caution when predicting distance from accelerometry derived metrics, accelerometers are also capable of identifying and quantifying a variety of locomotor events including steps, jumps, bounds, and impacts, among others. Since GNSS is limited to measures related to horizontal position (i.e., speed and distance), if accelerometers can also be used to estimate distance in mixed type activities such as sport, given their ability to quantify a variety of sport related movements, accelerometers may provide advantages compared to GNSS.

## Figures and Tables

**Table 1:** Dimensions of the small and large circles.

	Small Circle	Large Circle
Diameter	2 m	8 m
Circumference	6.28 m	25.13 m
Distance	2× laps = 12.56 m	Half-lap = 12.56 m

**Table 2:** Number of laps and distance traveled around the small and large circles.

Participants	Large Circle	Known Distance/m	Small Circle	Known Distance/m
1	0.5	12.57	60	376.99
2	1	25.13	58	364.43
3	1.5	37.70	56	351.86
4	2	50.27	54	339.29
5	2.5	62.83	52	326.73
6	3	75.40	50	314.16
7	3.5	87.96	48	301.59
8	4	100.53	46	289.03
9	4.5	113.10	44	276.46
10	5	125.66	42	263.89
11	5.5	138.23	40	251.33
12	6	150.80	38	238.76
13	6.5	163.36	36	226.19
14	7	175.93	34	213.63
15	7.5	188.50	32	201.06
16	8	201.06	30	188.50
17	8.5	213.63	28	175.93
18	9	226.19	26	163.36
19	9.5	238.76	24	150.80
20	10	251.33	22	138.23
21	10.5	263.89	20	125.66
22	11	276.46	18	113.10
23	11.5	289.03	16	100.53
24	12	301.59	14	87.96
25	12.5	314.16	12	75.40
26	13	326.73	10	62.83
27	13.5	339.29	8	50.27
28	14	351.86	6	37.70
29	14.5	364.42	4	25.13
30	15	376.99	2	12.57

**Table 3:** Formula for each accelerometry based metric.

Metric	Definition and Formula *
SUM	$SUM=\sum_{s=1}^{n}\sqrt{x_{s}^{2}}+\sqrt{y_{s}^{2}}+\sqrt{z_{s}^{2}}$
MAG	$MAG=\sum_{s=1}^{n}\sqrt{x_{s}^{2}+y_{s}^{2}+z_{s}^{2}}$
Impulse Load **	$IL=\sum_{s=1}^{n}\frac{\sqrt{x_{s}^{2}+y_{s}^{2}+z_{s}^{2}}}{9.8067}$
Player Load	$PL=\sum_{s=1}^{n}\frac{\sqrt{{\left(x_{s=i+1}-x_{s=i}\right)}^{2}+{\left(y_{s=i+1}-y_{s=i}\right)}^{2}+{\left(z_{s=i+1}-z_{s=i}\right)}^{2}}}{100}$

* In the formulas above, *x* = forward and backward acceleration, *y* = lateral acceleration and *z* = vertical acceleration. ** IL is propriety to the manufacturer and is only associated with locomotor events that are detected by Zephyr (e.g., walking, running, bounding, jumping).

**Table 4:** Summary of linear regression models.

Independent Variable	Small Circle				Large Circle			
	R	R^2^	RMSE	*p*	R	R^2^	RMSE	*p*
GNSSD	0.999	0.997	0.047	<0.001	0.999	0.999	0.027	<0.001
Impulse Load	0.960	0.922	0.242	<0.001	0.976	0.952	0.189	<0.001
MAG	0.992	0.983	0.112	<0.001	0.997	0.995	0.064	<0.001
SUM	0.992	0.984	0.109	<0.001	0.992	0.983	0.112	<0.001
Player Load	0.994	0.987	0.098	<0.001	0987	0.973	0.141	<0.001

**Table 5:** Details for each trial include laps and known distance completed around the small circle course, GNSS measured distance, and all accelerometry derived metrics.

Participant	Small Circle						
	Number of Laps	Known Distance/m	GNSSD/m	Impulse Load	MAG	SUM	Player Load
1	60	376.99	339.6	1234.76	848.23	57,277.7	17.36
2	58	364.43	322.7	1285.58	846.6	46,164.84	22.06
3	56	351.86	324.4	2018.93	1006.59	59,245.55	21.07
4	54	339.29	306.7	1584.19	868.55	45,124.71	19.57
5	52	326.73	277.1	1504.79	776.3	43,471.19	18.62
6	50	314.16	288.4	1835.24	976.83	46,687.57	19.11
7	48	301.59	229.4	987.58	698.06	40,605.42	18.43
8	46	289.03	263.3	644.4	541.58	43,342.69	17.32
9	44	276.46	242.3	1433.89	741.05	32,156.7	16
10	42	263.89	233.8	1108.26	636.97	32,585	12.68
11	40	251.33	229.8	1100.13	674.37	39,186.64	13.02
12	38	238.76	217.3	700.67	511.39	43,028.1	14.51
13	36	226.19	199.3	795.85	588.16	33,025.3	13.83
14	34	213.63	182.6	930.75	514.36	27,075.45	10.49
15	32	201.06	174.4	1373.54	551.03	29,721.93	12
16	30	188.5	176.1	797.74	457.17	25,862.01	10.09
17	28	175.93	149.6	961.1	435.61	20,382.67	9.64
18	26	163.36	149.6	583.73	407.22	22,577.47	7.82
19	24	150.8	135.2	479.04	306.43	20,562.16	8.15
20	22	138.23	125.5	725.24	385.17	19,100.84	8
21	20	125.66	110.2	385.47	250.08	16,287.71	8.58
22	18	113.1	99.7	491.59	285.11	15,775.49	6.05
23	16	100.53	90.1	399.75	241.1	16,023.39	5.35
24	14	87.96	78.2	355.25	196.18	10,961.84	4.77
25	12	75.4	61.1	328.71	174.47	8617.43	4.7
26	10	62.83	58.8	257.96	170.54	8207.35	4.06
27	8	50.27	46.7	193.38	128.41	7337.94	2.49
28	6	37.7	34.1	188.47	84.5	4987.59	1.86
29	4	25.13	20.3	117.37	63.33	2904.64	1.51
30	2	376.99	11.2	66.64	33.08	1916.23	0.67

**Table 6:** Details for each trial include laps and known distance completed around the large circle course, GNSS measured distance, and all accelerometry derived metrics.

Participant	Large Circle						
	Number of Laps	Known Distance/m	GNSSD/m	Impulse Load	MAG	SUM	Player Load
1	0.5	12.57	12.3	35.95	25.49	1615.13	0.48
2	1	25.13	25.2	103.53	57.66	2789.17	1.88
3	1.5	37.70	36.6	198.92	90.43	4607.63	2.59
4	2	50.27	45.5	274.39	117.24	5121.1	2.86
5	2.5	62.83	61.3	297.61	126.21	6540.61	3.2
6	3	75.40	75.2	229.65	172.02	8491.27	3.9
7	3.5	87.96	82.1	374.6	185.52	9026.94	5.23
8	4	100.53	97.8	390.72	213.75	9869.57	4.98
9	4.5	113.10	109.4	598.23	235.34	10,437.65	5.88
10	5	125.66	122.9	597.66	264.97	11,826.61	5.94
11	5.5	138.23	131.6	439.48	311.61	16,615.76	6.93
12	6	150.80	148.8	471.66	350.42	18,919.17	8.81
13	6.5	163.36	158.5	719.49	358.9	18,218.36	7.93
14	7	175.93	172.4	895.41	399.65	17,302.98	9.91
15	7.5	188.50	174	1071.26	418.64	23,473.34	10.5
16	8	201.06	192.5	999.68	437.84	22,546.53	9.62
17	8.5	213.63	205.1	1154.62	420.24	19,031.75	11.63
18	9	226.19	222.1	1006.09	488.78	24,166.76	10.03
19	9.5	238.76	233.9	819.64	514.5	27,886.33	11.93
20	10	251.33	249.4	1238.42	669.27	28,285.3	15.27
21	10.5	263.89	259.9	1215.66	560.68	26,430.16	16.72
22	11	276.46	273.9	981.49	648.91	32,951.12	13.32
23	11.5	289.03	283.3	1073.9	641.12	37,486.39	14.05
24	12	301.59	300.9	1081.76	646.01	31,967.92	15.82
25	12.5	314.16	288.1	1460.79	631.2	27,005.63	19.97
26	13	326.73	312.3	1364.23	680.51	32,644.37	17.12
27	13.5	339.29	338.1	1223.83	709.73	35,287.43	14.34
28	14	351.86	347.1	1833.86	767.71	35,025.29	18.75
29	14.5	364.42	344.6	1631.2	796.79	35,797.69	20.84
30	15	376.99	381.3	2339.7	881.78	45,740.11	20.47

## Data Availability

Not applicable.
